# Structure Formation in Antifriction Composites with a Nickel Matrix and Its Effect on Properties

**DOI:** 10.3390/ma15093404

**Published:** 2022-05-09

**Authors:** Kayode Olaleye, Tetiana Roik, Adam Kurzawa, Oleg Gavrysh, Iulia Vitsiuk, Krzysztof Jamroziak

**Affiliations:** 1 Department of Mechanics, Materials and Biomedical Engineering, Faculty of Mechanical Engineering, Wroclaw University of Science and Technology, 27 Wyspianskiego Str., 50-370 Wroclaw, Poland; krzysztof.jamroziak@pwr.edu.pl; 2 National Technical University of Ukraine “Igor Sikorsky Kyiv Polytechnic Institute”, 37 Peremogy Ave., 03057 Kyiv, Ukraine; roik2011@gmail.com (T.R.); gelo-1@ukr.net (O.G.); iuvitsiuk@gmail.com (I.V.); 3 Department of Lightweight Elements Engineering, Foundry and Automation, Wroclaw University of Science and Technology, 27 Wyspianskiego Str., 50-370 Wroclaw, Poland; adam.kurzawa@pwr.edu.pl

**Keywords:** powder, nickel alloy, antifriction composite, technology, alloying elements, structure, homogeneity, temperature, properties, friction units

## Abstract

The paper is devoted to studying the chemical elements distribution in the material’s structure depending on the manufacturing technological parameters and their effect on properties of a new self-lubricating antifriction composite based on powder nickel alloy EP975 with CaF_2_ solid lubricant for operation at temperature 800 °C and loads up to 5.0 MPa, in air. The study is focused on the features of alloying elements distribution in the composite matrix, which depends on the manufacturing technology. A uniform distribution of all alloying elements in the studied composite was shown. The chemical elements’ uniform distribution in the material is associated with one of the most important preparatory technological operations in the general manufacturing technology used. This is a technological operation of mixing powders with subsequent analysis of the finished mixture. The uniform distribution of chemical elements determines the uniform arrangement of carbides and intermetallics in the composite. General manufacturing technology, which includes the main operations, such as hot isostatic pressing technology and hardening heat treatment, contributed to the obtainment of a practically isotropic composite with almost the same properties in the longitudinal and transverse directions. Because of the composite’s structural homogeneity, without texturing, characteristics are isotropic. Improving the material’s structural homogeneity helps to keep its mechanical and anti-friction qualities stable at high temperatures and stresses in the air. The performed studies demonstrated the correctness of the developed manufacturing technology that was confirmed by the electron microscopy method, micro-X-ray spectral analysis, mechanical and tribological tests. The developed high-temperature antifriction composite can be recommended for severe operating conditions, such as friction units of turbines, gas pumping stations, and high-temperature units of foundry metallurgical equipment.

## 1. Introduction

A necessary condition for the stable operation of machines and equipment is the use of contact pair materials, primarily antifriction materials. The work of any antifriction material is due to its properties and depends on the working conditions, such as pressure, temperature, speed, and influence of the environment. This is especially important for antifriction materials exposed to extreme conditions—high loads, aggressive environments, and elevated and high temperatures more than 600 °C. According to the authors [1,2,3], 80% of all failures of metal assemblies are due to friction and wear. The advent of new technologies and production processes along with the need to increase the lifetime of friction units has led to the need to increase the life of antifriction components. Due to their high mechanical qualities, wear resistance, and low thermal expansion coefficient, metal matrix composites (MMCs) have been widely employed in numerous areas such as the car industry, nuclear power industry, and aerospace industry [4,5]. As the operating temperature of sophisticated engines rises, the parts must withstand high temperatures and substantial temperature variations (start-stop and run), whereas most machine parts must function under complicated stress for an extended period. The strength and lubrication of moving components has become a critical aspect determining the whole system’s dependability and longevity [6,7,8]. Thus, the high-quality standards of the industry working with high-temperature branches require predictability and updated data to develop antifriction elements that can work for a longer service life compared to the known [3,9,10]. Among the known antifriction materials, a separate group consists of materials designed for severe conditions, characterized by high temperatures (700–800 °C) with simultaneous action of high loads (5–8 MPa) in the air. Cobalt or nickel-based materials are widely used for these operating conditions [3]. Known cast and powder nickel-based antifriction materials have high mechanical properties, high heat resistance, but they do not satisfy the operational requirements due to the high values of friction coefficient and wear rate at 700–800 °C in air [1,2,11,12,13,14,15].

Damages in antifriction materials subjected to high temperature and load are of great problem in many industrial fields, for example in power engineering industry. Therefore, antifriction composite materials include anti-adhesive additives that can provide long life of friction units operating under severe operating conditions [1,2,15,16]. This is due to the inability to use liquid oils at high operating temperatures. Iron-based materials can no longer keep up with the demands of contemporary machinery. In several situations, cast materials exhibit insufficient performance qualities (high friction coefficient and wear) or are entirely nonfunctional. Moreover, cast materials are prohibited from containing a variety of chemicals. Current powder materials are devoid of these flaws, but they are costly because to the high cost of raw ingredients [15,16,17]. For example, the known Ni composites have alloying elements, such as Cr and V, which allow the obtainment of materials with high mechanical properties. These materials are capable to operate at high temperatures (up to 600 °C), maintaining their structural strength [1,2,6]. In different cases, molybdenum dioxide MoS_2_ was used as solid lubricant. The presence of solid lubricant MoS_2_ allows working in self-lubricating mode at high loads and temperatures up to 600 °C. In this case, the friction coefficient f is 0.5, while the condition of high antifriction is f < 0.3 [1,2,6]. Temperature > 600 °C leads to the significant heating of the contact surfaces for such materials. Molybdenum dioxide MoS_2_ dissociates and forms the atomic Mo, which is instantly oxidized to form MoO_3_ oxide in the air. The composite material oxidation extends to the depth, resulting in the destruction of such composites [6]. Other antifriction Ni composites contain reinforced fibers, which significantly increase such materials heat resistance; however, the antifriction characteristics remain unsatisfactory at high temperatures up to 700–800 °C [1,2,6].

The authors of [11,12,13,14,15] proposed composite materials based on nickel, which showed satisfactory results. However, they showed unsatisfactory tribological properties at temperatures above 600 °C. Recently, new high-alloy Ni antifriction composites have been developed for temperatures up to 700 °C [1,16]. These composites showed high and stable antifriction properties.

The microstructure and mechanical characteristics of a chilled composite made of nickel matrix and SiO_2_ particles as matrix reinforcement were examined and assessed by the authors. Using a stir casting process with various cold materials and reinforced content, the author has effectively manufactured Nickel based matrix composites from a typical electric induction furnace. The microstructure of chilled composite is finer than that of unchilled matrix alloy, according to the authors of [18,19,20]. However, features of the structure and the nature of the alloying elements distribution in the structure leading to a high level of properties have not been studied yet. This is because the structure’s evolution is the main factor determining the properties of the composite, depending on the manufacturing technology. These arguments were the basis for research in this direction. Therefore, the study of the nature of alloying elements distribution, their influence on the structural features, and properties of high temperature antifriction composites is a very relevant problem which requires further research. The combinatorial impact of solid lubricant on lump form composites has also been widely studied [21,22,23]. High-strength intermetallic Ni-Al [24,25,26], Ti-Al [27], and Fe-Al [28] matrix composites with solid lubricants, such as Ag/BaF_2_CaF_2_, Ag/BaCrO_4_, Ag/Ti_3_SiC_2_, MoS_2_/BN/Ti_3_SiC_2_, and Ba0.25Sr0.75SO_4_, were created by hot pressed sintering and spark plasma sintering. Furthermore, from RT to 1000 °C, the ZrO_2_ [29] ceramic matrix composite including MoS_2_/CaF_2_ showed high self-lubricity. Furthermore, the Ti_6_Al_4_V and NiCr alloy matrix composites, which used Ag/MoO_3_, Ag/BaF_2_/CaF_2_, SrSO_4_, CaF_2_, and other solid lubricants as solid lubricants, demonstrated good lubricating capabilities from room temperature to 900 °C [30,31,32,33,34,35]. The solution of this scientific problem opens ways for obtaining highly effective composite tribological materials with controlled structure and predicted high functional properties. This is especially important for composites operating at high temperatures, when it is necessary to rationally use high-temperature solid lubricants to ensure their stable lubricating action under extreme conditions. For these purposes, effective solid lubricants are the class of alkaline earth metal fluorides, such as BaF_2_, CaF_2_, AlF_3_, and MgF_2_ [1,2,4,6,7,8,9,10,14,21,29].

It should be noted the use of a base material for antifriction parts from alloyed powder raw materials ensures the formation of a more homogeneous structure compared to the structure formed as a result of the alloying elements separate addition to the base matrix. For example, the authors of [1,2,10] found the plasticity of composites obtained from alloyed powders is 3–4 times higher than composites manufactured from the pure metal powders mixture.

Therefore, the use of alloyed raw materials as the basis for composites intended for severe operating conditions is undoubted.

Nevertheless, a number of issues related to the distribution of alloying elements and its influence on the properties of highly alloyed antifriction nickel composites remain unexplored.

The objective of this article is to study the chemical elements distribution in the structure of material depending on the manufacturing technological parameters and their effect on properties of the developed self-lubricating nickel-based antifriction composite in the system “high-alloyed Ni-alloy—CaF_2_ solid lubricant” designed to operate at 800 °C on air.

## 2. Materials and Methods

Chemical elements’ distribution in the structure was studied using raster electron microscope; calcium fluoride solid lubricant in the composite was identified using scanning electron microscopy (SEM). Micro-X-ray spectral analysis was carried out using a raster electron microscope. For comparative tests, samples were made from the studied composite based on EP975 powder nickel alloy and known powder material based on Ni in the amount of 20 pieces of each material. All mechanical tests were carried out according to standard methods by ASTM D7264, ISO 6506/ASTM E10. Measurements of the developed material’s density and the compared Ni-composite were performed according to the standard method according to the standard ISO2738:1999 for sintered materials.

Comparative tribological tests were performed on a VMT-1 friction testing high-temperature machine at temperature up to 800 °C, sliding speed V = 1.0 m/s and load up to P = 5.0 MPa, the counterface is made of stainless steel EI961Sh. This EI961Sh steel corresponded to the material of the real shafts in the high-temperature friction units in power engineering equipment. The EI961Sh steel’s chemical composition has been presented in Table 1. Tribological tests were performed according to the end-friction scheme; the friction track was 5 km.

The study focused on new antifriction composite materials based on powder nickel alloy EP975. The powder Ni-alloy EP975 was the basis for new composites. Powders of the high-alloyed nickel alloy EP975 were produced by powder spraying method of melted metal by argon stream. These sprayed powders are the industrial standard powders for the manufacture of different high temperature heat resistant parts. Such sprayed powders are the spherical particles after industrial production. Additional preparatory operations with the EP975 powder alloy were not carried out. In our experiments, the powders 60–240 µm in dimension were used. As solid lubricant powder of calcium fluoride (CaF_2_) was added to the original charge. CaF_2_ powders were dried for 1 h at 100 °C and sifted through a sieve to obtain the powder fraction of 125 µm. Such heterogeneity of the initial powders is a favorable factor for the fabrication of dense composites [2].

This CaF_2_ solid lubricant CaF_2_ is effective at high temperatures and retains its properties up to 1300 °C. [1,2,15,16,17,18,19,20,21,22,23]. Thus, we studied a self-lubricating nickel-based antifriction composite, which is a system of high-alloyed Ni-alloy EP975 + (4.0–8.0)% CaF_2_. Chemical composition of the researched composite has been presented in Table 2 [1].

As it can be seen from Table 2, powder nickel alloy EP975 contains many alloying elements. Therefore, it is a very important circumstance to study the distribution of these elements in the material’s structure, their influence on structure features, on which the properties of the composite depend.

The studied composites were produced by hot isostatic pressing technology (HIP) because the traditional powder metallurgy technology doesn’t ensure minimum porosity [1,6,16].

HIP technology combines the forming and sintering processes due to simultaneous action of high pressure (all-round compression) and high temperature [1,6,16]. This HIP process’s feature is maximum compaction and consolidation of the composite in 1 stage.

To prepare the initial charge the sprayed nickel alloy EP975 powders and solid lubricant (CaF_2_) were mixed up during 5–6 h with subsequent analysis of the finished mixture. This technological operation is very necessary to avoid segregation by component density. Then the initial powder mixture is freely poured into a container; the container is installed in the HIP machine and subjected to HIP process. The hot isostatic pressing process was carried out at 1210 ± 10 °C, during 4–5 h, under pressure 130–140 MPa. Such technology allows obtaining a practically non-porous composite. As a result, the blanks had a relative density ≈ 99.9% after using HIP technology. This is especially important fact for the material working at high temperatures (up to 800 °C) in an oxidizing environment, when porosity is unacceptable.

Moreover, HIP technology provides an isotropy of material having the same properties in all three directions due to the effect of all-round compression in the HIP process. Further, a heat treatment was performed to isolate the excess hardening phases in the metal nickel matrix of the composite. The heat treatment parameters were as follows: hardening with heating up to 1240–1250 °C and cooling on air, and then aging at 920 °C for 15–16 h.

## 3. Results and Discussion

**Figure 1:**
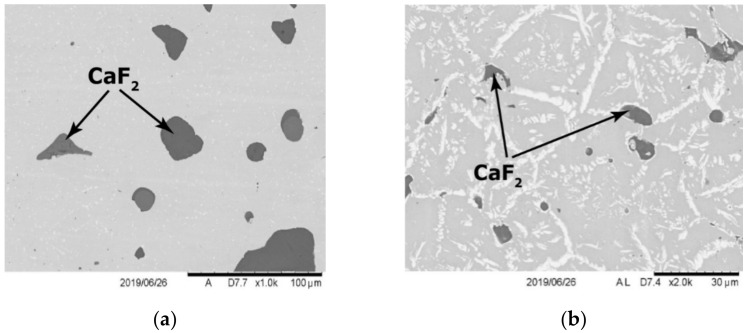
Structure of the material, wt.%: EP975 + 6CaF_2_; (**a**) non-etched section; (**b**) etched section.

**Figure 2:**
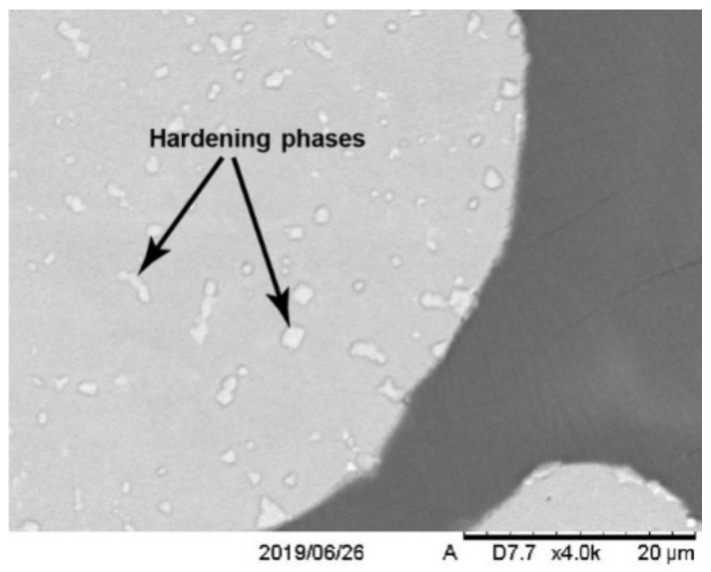
Hardening phases in a metal matrix.

The structure of the material was formed after the described technological operations and consisted of a metal matrix and CaF_2_ solid lubricant inclusions (Figure 1). Metal matrix represents a γ solid solution of alloy elements in nickel strengthened with intermetallics and carbides of alloying elements (Figure 2).

As it can be seen from Figure 1 and Figure 2, the hardening phases are uniformly distributed in the nickel matrix of the composite. To study such morphological features of the structure, fine studies of the alloying elements distribution in the matrix were carried out.

The alloying elements’ distribution in the EP975 nickel powder alloy is directly related to the technology of its manufacture, namely, to melt spraying.

However, the degree of homogeneity in the distribution of these alloying elements remains unexplored after using such a harsh hot isostatic pressing technology. Therefore, it is very important to know the distribution of elements in the finished composite after HIP.

**Figure 3:**
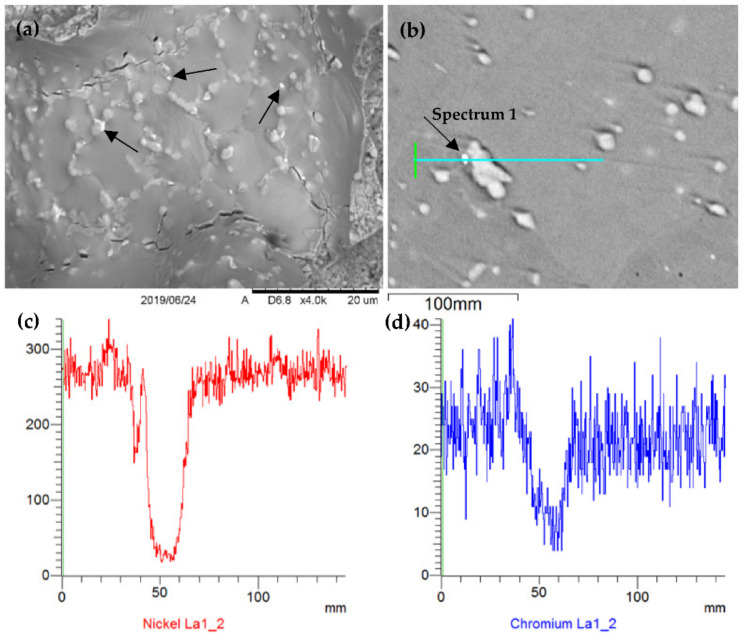
SEM analysis of alloying elements in a composite’s material: (**a**) fracture, Ni_2_Cr -phase is shown by arrows; (**b**–**d**) EDS linear analysis results.

**Figure 4:**
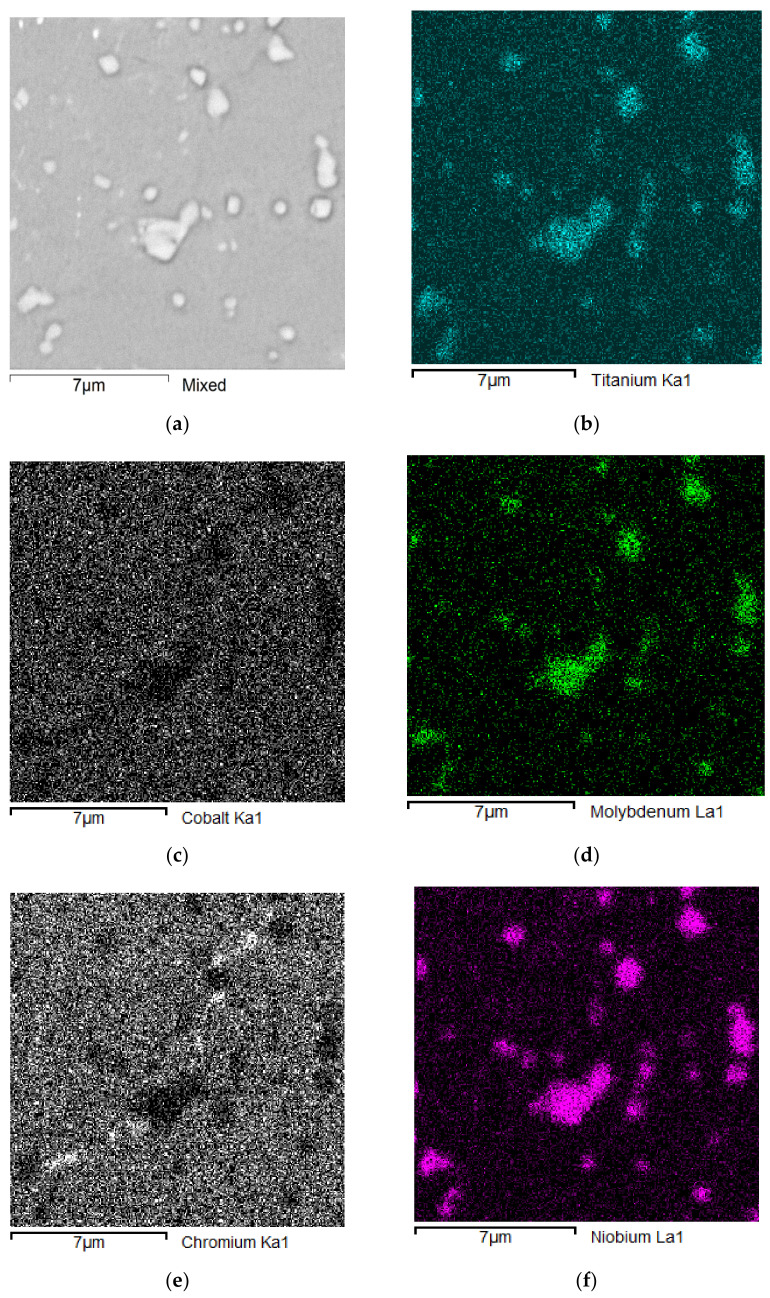

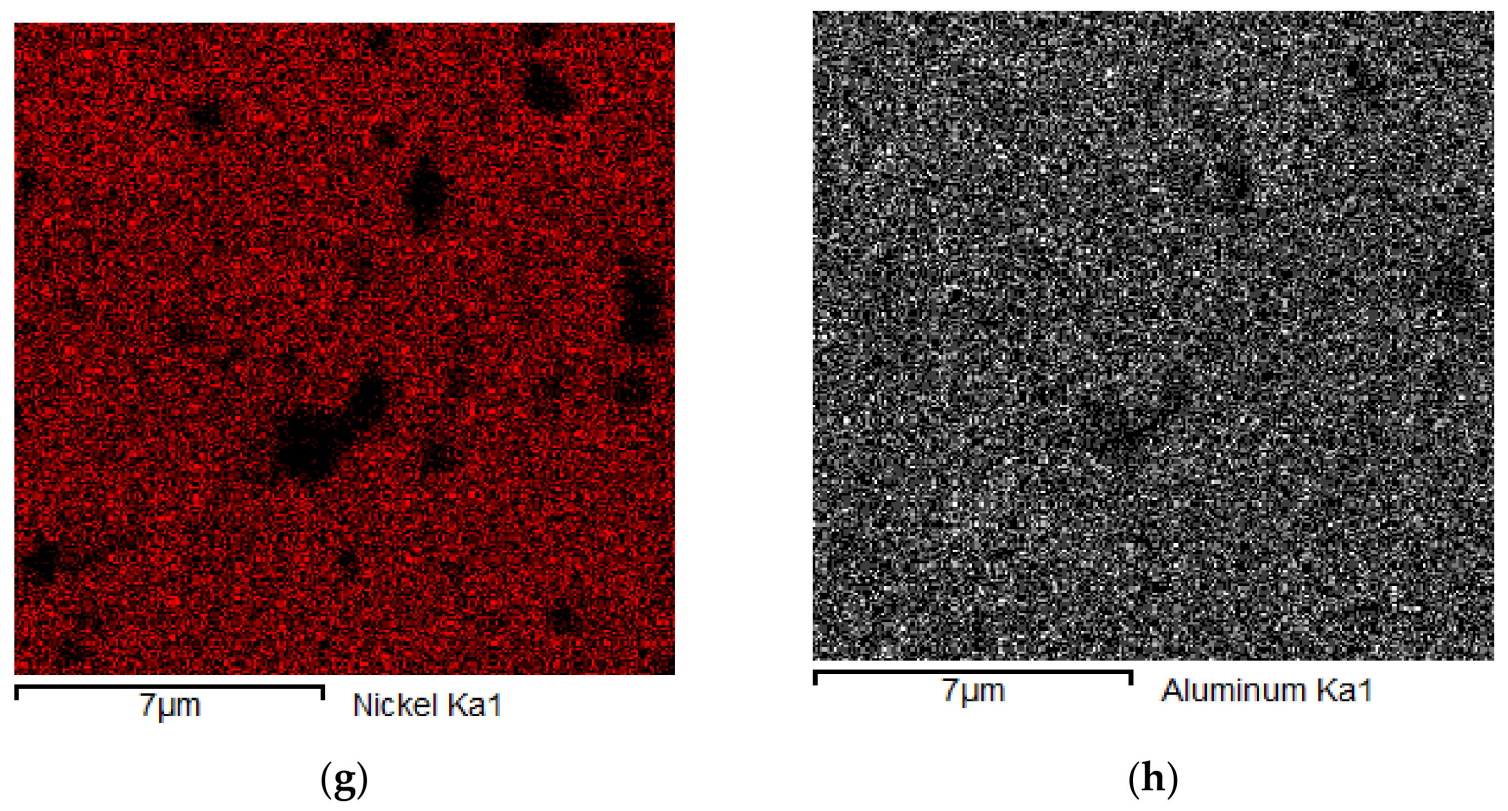
Maps of elements distribution in the composite’s structure, analysis parameters: Acc. Voltage: 15.0 kV; Resolution: 512 × 512 pixels; Viewed Resolution: 50%; Process Time: 5 s; Image Width: 14.7 µm. (**a**) Mix; (**b**) Titanium; (**c**) Cobalt; (**d**) Molybdenum; (**e**) Chromium; (**f**) Niobium; (**g**) Nickel; (**h**) Aluminum.

For this purpose, maps of elements distribution in the composite’s structure were obtained (Figure 3 and Figure 4). The conducted EDS analysis and distribution maps of alloying elements in the material showed that there is a mutual correlation between the alloying elements and corresponding phases of the composite.

Thus, during the formation process, chemical elements interact in two basic systems: Ni-W-Co-Ti-Cr-Al and Ti-Nb-Mo. The complex system based on nickel Ni is the basic system forming the matrix of the composite.

Probably the hardening phases of the Ti-Nb-Mo system are also present in the composite, as indicated by small accumulations in Figure 2. It is known [36,37] that in the Ti-Nb-Mo triple system, the formation of TiNbMo phase is possible during the alloy fabrication. This phase represents a β-solid solution and could have been formed during the fabrication of the highly alloyed sputtered powder EP975 nickel alloy, which contains a significant amount of Ti, Nb, and Mo (Table 3). The formed β-phase has high strength and corrosion resistance in accordance with the data [36,37]. This is a favorable circumstance for the use of the studied composite based on the EP975 nickel powder alloy under severe operating conditions. The study of β-phase has been presented below (structural and SEM analysis).

It is known [38,39] that in the Ni-Cr system the existence of an intermediate δ-phase (Ni_2_Cr) is possible at temperatures below 600 °C, which is formed in the solid state. Experimental studies and microscopic observations also confirm that the δ-phase Ni_2_Cr is present around the boundaries in trace amounts (Figure 3a). EDS identification of alloying elements and the distribution of the δ-phase have been presented in Figure 3 and Table 3.

The results of structural and SEM analyses (Figure 3, Table 3) convincingly showed the presence of the δ -phase in the studied composite. Moreover, as can be seen from Figure 3a, the distribution of this phase is uniform throughout the volume of the material. 

To study the complete picture of the alloying elements distribution in the composite’s structure, maps of the elements distribution were obtained (Figure 4). Analyses carried out on all tested samples confirmed the high repeatability of the obtained chemical composition results (Figure 4). This is due to the correctness of the used technological modes of manufacture, which ensured the homogeneity of the alloying elements distribution and the absence of segregation phenomena.

**Figure 5:**
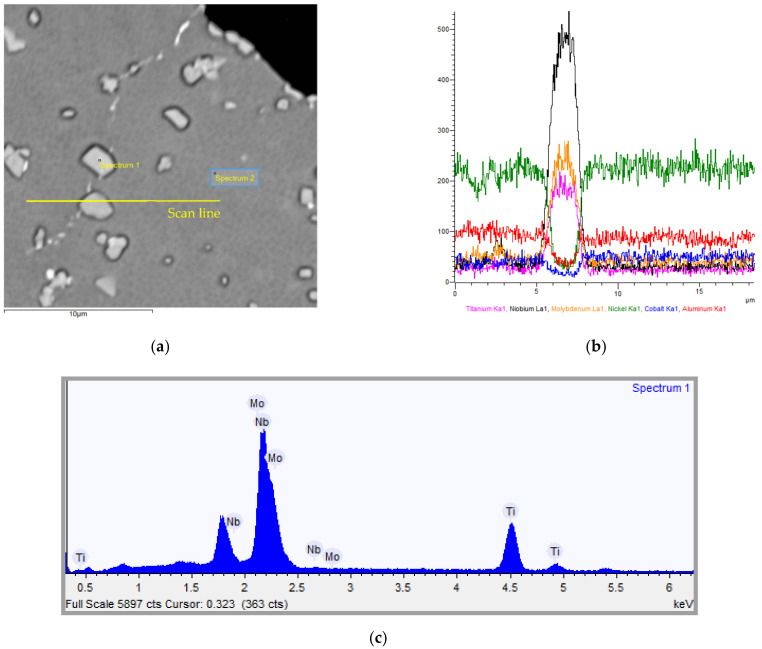

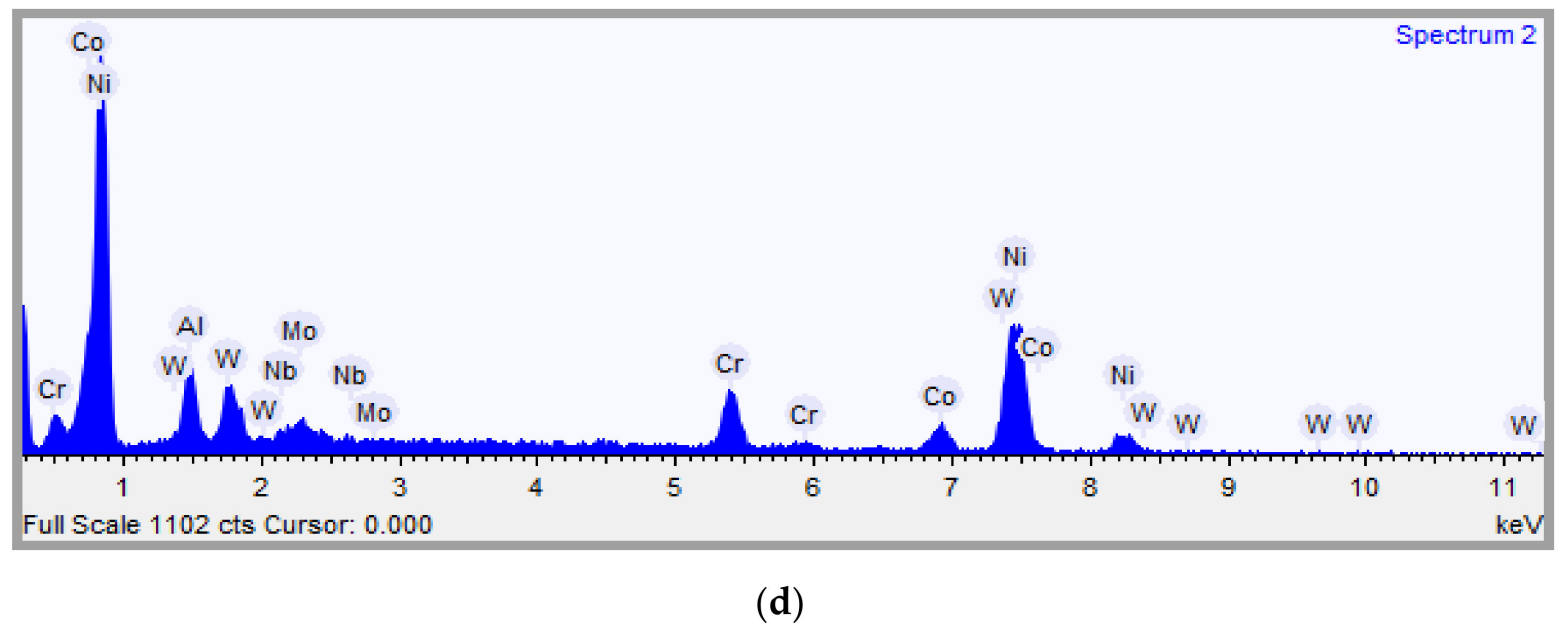
SEM analysis of alloying elements in a composite’s matrix: (**a**) SEM: microstructure of the material, β-phase inclusions; (**b**)linear analysis results, (**c**) Spectrum 1, (**d**) Spectrum 2.

It should be noted that X-ray microanalysis made it possible to determine the concentration ratio of chemical elements in the composite’s matrix (Figure 5) and hardening phases, so the β-phase located mainly at the grain boundaries (Figure 5a, Table 4 and Table 5).

The results of micro-X-ray spectral analysis (Figure 5, Table 5) showed the participation of powder EP975 nickel alloy’s chemical elements in the formation of the hardening phases in the studied composite (Figure 2).

In addition to the significant influence of manufacturing technology, the uniform distribution of chemical elements provides the same uniform distribution of the hardening phases in the areas corresponding to these elements. The presence of hardening phases contributes the increase in mechanical properties and directly effects the tribological characteristics (Table 6).

Table 6 demonstrates comparative performance for composites operating under high temperature friction conditions. As it can be seen from Table 6, the HIP technology made it possible to obtain a dense, almost compact material, which had a favorable effect on its properties, both mechanical and tribological compared to known highly porous Ni-based composite, obtained by the traditional technology of powder metallurgy [1,2,6]. This composite has a porosity ≈ 14%, determined according to the standard ISO2738:1999-Sintered metal materials, excluding hardmetals—permeable sintered metal materials—determination of density, oil content, and open porosity.

Moreover, manufacturing technology, including HIP technology and hardening heat treatment, contributed to the production of a practically isotropic composite. Anisotropy of properties is completely absent, as it can be seen from Table 7.

Table 7 shows that the studied composite based on EP975 powder nickel alloy demonstrates almost the same properties in the longitudinal and transverse directions, in contrast to the known Ni-composite [1,2,6] obtained by traditional powder metallurgy technology. An analysis of the mechanical properties (Table 7) indicates that the studied composite based on EP975 powder Ni alloy is an isotropic material. 

Unlike the composite EP975 + (4–8)% CaF_2_, the known powder material based on Ni [1,2,6] is anisotropic. Its mechanical properties are significantly different in the longitudinal and transverse directions. This is due to the manufacturing technology, when the traditional pressing process takes place in the longitudinal direction.

Therefore, the properties of the known Ni composite [1,2,6] in the longitudinal direction are higher than in the transverse direction. The traditional powder technology for its manufacture gives a reason to conclude that such a composite has a strong texture, and, as a result, properties anisotropy. Moreover, the known powder material has a porosity of about 14%, in contrast to the studied composite, which is practically pore-free after using HIP technology.

**Figure 6:**
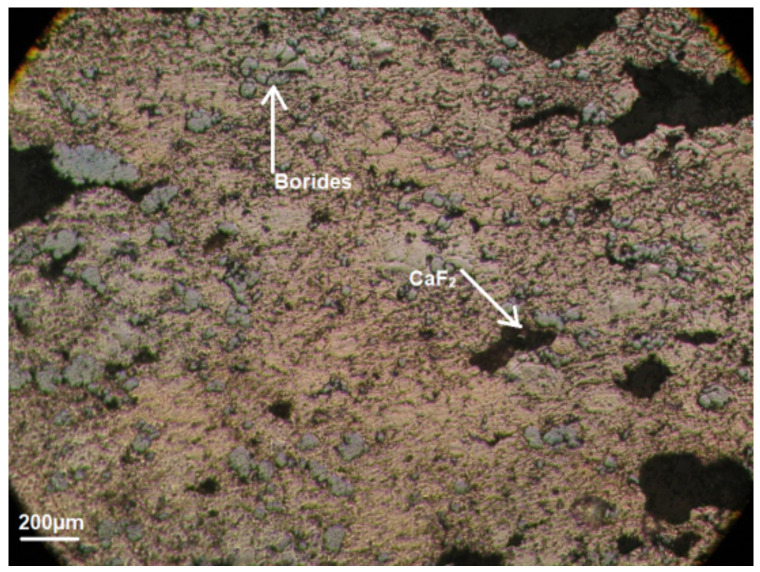
Structure of the Ni + (18–45)%(MoB_2_ + ZrB_2_) + 5% (CaF_2_ or BaF_2_) composite material.

In addition, this composite [1,2,6] has an uneven distribution of components (borides and fluorides) in the structure (Figure 6).

Figure 6 also shows the elongation of the material components, where the pressing pressure was directed. This indicates the texture of this composite.

Such a fact as structural heterogeneity in the distribution of components also affected the level of properties (Table 7) in addition to technological factors for the known Ni + (18–45)% (MoB_2_ + ZrB_2_) + 5% (CaF_2_ or BaF_2_) composite material [1,2,6].

Isotropy of the EP975 + (4–8)% CaF_2_ composite’s properties can be explained by two factors. First of all, this is a consequence of the hot isostatic pressing (HIP) technology used, when the initial powder mixture is subjected to all-around compression at high pressure with simultaneous action of high temperature. As a result, complete consolidation of the material is achieved. The second, no less important factor is the homogeneity of the alloying elements distribution, which is confirmed by spectral analysis. The isotropy of the studied material’s properties (Table 7) is a consequence of the composite’s structural homogeneity, which directly depends on the chemical element’s uniform distribution and the segregation phenomena absence. Uniformity in the distribution of alloying elements is a prerequisite for the formation of corresponding hardening phases in these places.

**Figure 7:**
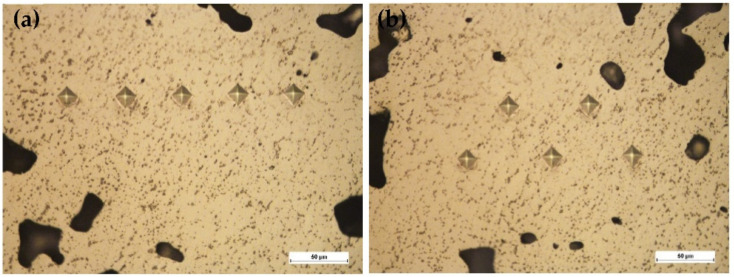
Image of the composite’s structure, wt.%: EP975 + 6CaF_2_ with marked imprints of the HV0.1 microhardness measurement: (**a**) Area 1, (**b**) Area 4.

In order to confirm the homogeneity of the developed composite materials, additional microhardness tests were performed. For this purpose, some areas were selected in the material’s volume, where the microhardness measurements were made. The results have been shown in Figure 7 and Table 8.

Figure 7 and Table 8 showed that the microhardness tests confirmed the high reproducibility of the results which carried out in different places of the tested samples.

This indicates a high homogeneity of the studied composite material based on EP975 powder Ni alloy. The microhardness average value ranged from 495HV0.1 to 507HV0.1 over the entire volume of the composite.

Thus, improving the material’s structural homogeneity contributes to the stabilization of its mechanical and antifriction properties (Table 6) at high operating temperatures and loads in the air.

## 4. Conclusions

- For the first time, a comprehensive study was performed on the manufacturing technology effect on the formation of the EP975 + (4–8)% CaF_2_ antifriction composite’s an isotropic structure and properties. We have studied the structural features and alloying elements distribution in the new effective composite antifriction material based on powder Ni alloy EP975 with solid lubricant CaF_2_.
- The developed antifriction composite demonstrates high mechanical and tribological properties and performs well in more severe conditions than known Ni composite material. The mechanical properties of the studied composite are 3.0–3.5 times higher than those of the known Ni powder composite. The composite’s structure substantially effects on the tribological characteristics and determine its behavior in high-temperature friction unit. The developed antifriction composite has a friction coefficient approximately 1.5 times lower than that of the known composite, and wear resistance is more than 7–8 times higher at temperature up to 800 °C. These differences are associated with significant differences in their manufacturing technologies, which lead to differences in their structure’s formation and properties.
- For the first time, it has been shown the elemental homogeneity throughout the composite’s entire volume contributes to the formation of a homogeneous structure, completely excluding segregation. This, in turn, ensures the isotropy of the studied composite’s properties.
- The performed studies demonstrated the correctness of the developed manufacturing technology that was confirmed by the electron microscopy method, micro-X-ray spectral analysis, mechanical and tribological tests. General manufacturing technology, which includes the main operations, such as hot isostatic pressing technology and hardening heat treatment, contributed to the obtaining of a practically isotropic composite with almost the same properties in the longitudinal and transverse directions. The composite is isotropic in its characteristics due to structural homogeneity, without texturing.
- The research results make it possible to recommend the studied antifriction composite for severe operating conditions, such as friction units of turbines, gas pumping stations, and high-temperature antifriction units of foundry metallurgical equipment.

## Figures and Tables

**Table 1:** Chemical Composition of the Stainless Steel EI961Sh Counterface.

Components, wt.%										
Carbon	Tungsten	Chromium	Molybdenum	Vanadium	Silicon	Nickel	Manganese	Sulfur	Phosphorus	Iron
0.10–0.16	1.60–2.00	10.5–12.0	0.35–0.50	0.18–0.30	to 0.6	1.50–1.80	to 0.6	to 0.025	to 0.030	basis

**Table 2:** Chemical Composition of the Materials Based on Powder Nickel Alloy EP975.

Components, wt.%									
Carbon	Tungsten	Chromium	Molybdenum	Titanium	Aluminium	Niobium	Cobalt	Nickel	Calcium Fluoride
0.038–0.076	8.65–9.31	7.6–9.5	2.28–3.04	1.71–2.09	4.75–5.13	1.71–2.59	9.5–11.4	basis	4.0–8.0

**Table 3:** Spectrum1: Concentration Ratio of Alloying Elements in a δ-phase (Ni_2_Cr) Phase.

Element	Cr	Ni
Weight %	33.382	66.618
Atomic %	36.135	63.865

**Table 4:** Spectrum1: Concentration Ratio of Alloying Elements in a Composite’s Phase.

Element	Ti	Nb	Mo
Weight %	27.215	65.126	7.658
Atomic %	42.118	51.965	5.917

**Table 5:** Spectrum2: Concentration Ratio of Alloying Elements in a Composite’s Matrix.

Element	C	O	Al	Ti	Cr	Mo	Co	Nb	W	Ni
Weight %	0.048	0.118	4.361	1.416	9.974	3.535	9.083	5.084	9.770	56.611
Atomic %	0.145	0.372	7.460	1.364	8.966	4.442	8.330	4.532	8.261	56.128

**Table 6:** Mechanical and Tribological Properties of the Materials.

Composition, wt. %	Porosity, %	Tensile Strength, σ_t_, MPa	Charpy Impact Strength, KC, J/m^2^	Hardness HB, MPa	Friction Coefficient/ Wear Rate, µm/km (at 5 MPa and 800 °C)	Maximum Allowable Load, MPa/ Temperature, °C
EP975 + (4–8) CaF_2_	0.1–0.11	1010–1170	520–650	2530–2610	(0.23–0.25)/(41–45)	5/800
Ni + (18–45) (MoB_2_ + ZrB_2_) + 5 (CaF_2_ or BaF_2_) composite material [1,2,6]	13–15	240–310	350–520	850–950	0.34/368	1.5/550

**Table 7:** Comparative Mechanical Properties in Longitudinal and Transverse Directions for the Studied and Known Composites.

Composition, wt. %	Relative Elongation, δ, %, Test Direction		Relative Narrowing, ψ, %, Test Direction		Tensile Strength, σ_t_, MPa Test Direction		Hardness, HB, MPa, Test Direction	
	Longitudinal	Transverse	Longitudinal	Transverse	Longitudinal	Transverse	Longitudinal	Transverse
EP975 + (4–8) CaF_2_	9.6–10.3	9.8–10.2	11.9–12.6	11.6–12.4	1010–1170	1005–1165	2550–2600	2530–2590
Ni + (18–45) (MoB_2_ + ZrB_2_) + 5 (CaF_2_ or BaF_2_) composite material [1,2,6]	4.1–4.3	2.7–2.9	5.7–6.0	4.4–4.7	240–310	170–190	850–950	780–790

**Table 8:** Results of microhardness HV0.1 measurements in different areas of samples.

Area	Microhardness HV0.1					
	Test 1	Test 2	Test 3	Test 4	Test 5	Average
Area 1	510	516	497	504	510	507
Area 2	495	510	501	497	512	503
Area 3	510	514	479	520	506	506
Area 4	516	493	519	504	501	507
Area 5	446	512	499	516	504	495

## Data Availability

Data sharing is not applicable to this article.
